# Classification of malignant tumours in breast ultrasound using unsupervised machine learning approaches

**DOI:** 10.1038/s41598-021-81008-x

**Published:** 2021-01-14

**Authors:** Wei-Chung Shia, Li-Sheng Lin, Dar-Ren Chen

**Affiliations:** 1 Molecular Medicine Laboratory, Department of Research, Changhua Christian Hospital, Changhua, Taiwan; 2 Department of Breast Surgery, The Affiliated Hospital (Group) of Putian University, Putian, Fujian China; 3 Comprehensive Breast Cancer Center, Changhua Christian Hospital, Changhua, Taiwan

**Keywords:** Breast cancer, Medical imaging

## Abstract

Traditional computer-aided diagnosis (CAD) processes include feature extraction, selection, and classification. Effective feature extraction in CAD is important in improving the classification’s performance. We introduce a machine-learning method and have designed an analysis procedure of benign and malignant breast tumour classification in ultrasound (US) images without a need for a priori tumour region-selection processing, thereby decreasing clinical diagnosis efforts while maintaining high classification performance. Our dataset constituted 677 US images (benign: 312, malignant: 365). Regarding two-dimensional US images, the oriented gradient descriptors’ histogram pyramid was extracted and utilised to obtain feature vectors. The correlation-based feature selection method was used to evaluate and select significant feature sets for further classification. Sequential minimal optimisation—combining local weight learning—was utilised for classification and performance enhancement. The image dataset’s classification performance showed an 81.64% sensitivity and 87.76% specificity for malignant images (area under the curve = 0.847). The positive and negative predictive values were 84.1 and 85.8%, respectively. Here, a new workflow, utilising machine learning to recognise malignant US images was proposed. Comparison of physician diagnoses and the automatic classifications made using machine learning yielded similar outcomes. This indicates the potential applicability of machine learning in clinical diagnoses.

## Introduction

Breast ultrasound (US) is an important non-radiation imaging method used to detect and classify breast tumours. It is well tolerated by patients and can be easily integrated into interventional procedures for patient treatments^1^. However, the accuracy of breast US is limited and depends on the experience and technical ability of the operator. Thus, US assessments show the inherent limitations associated with operator-dependent outcomes. Differences between operators, especially the divergence in their skill, knowledge, and understanding of various breast US techniques can lead to observer variations in the diagnosis.

For the improvement of risk assessment and quality of care, the Breast Imaging Reporting and Data System (BI-RADS)^2^ provides standardised terms for describing breast mass features and assessments in radiology, including mammography, magnetic resonance imaging (MRI), and US. This approach has been proven to be effective to distinguish between benign and malignant masses^3^. However, many US features in BI-RADS are associated with both malignant and benign masses. For category 4 breast masses, it is common to have both malignant and benign features in the report at the same time. Due to the wide range of malignancy risks in category 4 breast lesions (3–94%), the reproducibility among radiologists in the classification of subcategories 4A, 4B, and 4C is poor^4^.

Computer-aided diagnosis (CAD) uses a computerised program to assist the radiologist with image interpretation and diagnosis by providing a second objective opinion^5^. To improve diagnostic accuracy and reduce differences among observers, CAD systems have been used to distinguish between malignant and benign masses in ultrasound images of breast cancers^6,7^. Previous studies showed that the various CAD systems used in breast US imaging exhibited good diagnostic performance and decreased variability among observers^7^.

Traditional CAD processes include feature extraction, selection, and classification^8,9^. Having an effective strategy in feature extraction can improve overall performance^10^. However, the selection and extraction of meaningful image features from a dataset is a complicated and time-consuming task, which requires many pre-processing procedures and is usually heavily dependent on human effort. The inherent noise and speckle in ultrasound imaging and the use of various algorithms make fine-tuning of the overall performance of traditional CAD more difficult.

Thus, the aims of this study were to (a) increase the diagnostic performance associated with the classification of malignant tumours belonging to BI-RADS category 4 in US images, and (b) achieve comparable performance to those reported for deep learning techniques that are based on the cooperation of several machine learning algorithms.

## Methods

### Participants and data acquisition

This cross-sectional retrospective study was approved by the Institutional Review Board of Changhua Christian Hospital, Changhua, Taiwan (No. 181235). The requirement for informed consent was waived by the ethics committee because of the study’s retrospective nature. All experimental methods were supervised by the IRB and conducted in accordance with relevant guidelines and the Declaration of Helsinki (DOH).

The images were collected from 1 January 2017 to 31 December 2018. In total, 370 benign and 418 malignant masses were screened, and 677 patients were enrolled in this study. The exclusion criteria for patients with benign tumours included tissue types that were associated with the following conditions: inflammation (including autoinflammation, chronic inflammation, and xanthogranulomatous inflammation), abscesses, and spongiotic dermatitis. For patients with malignant tumours, the exclusion criteria included cases with unknown tissue types (or incomplete recordings), unknown BI-RADS category classification (undocumented), or incomplete US image reports. The patients’ ages ranged from 35 to 75 years. The US images captured the full-view of the screen (but did not include the text title, indicators, and relevant marks, etc.) with no markers of preselected tumour regions. This minimised the human effort required for image analysis and also provided detailed information to the image processing application. For each participant, at least two different scan planes of the tumour or solid masses were acquired in the US images. The diameter of the tumour was measured as the largest diameter of the tumour. The sonograms were acquired using GE Voluson 700 (GE Healthcare, Zipf, Austria) and Toshiba Aplio 500 (Toshiba Medical Systems Corporation, Otawara, Japan) ultrasound systems. During image acquisition, the patients were in a supine position with their arms extended over their heads. No acoustic stand-off pad was used.

**Figure 1:**
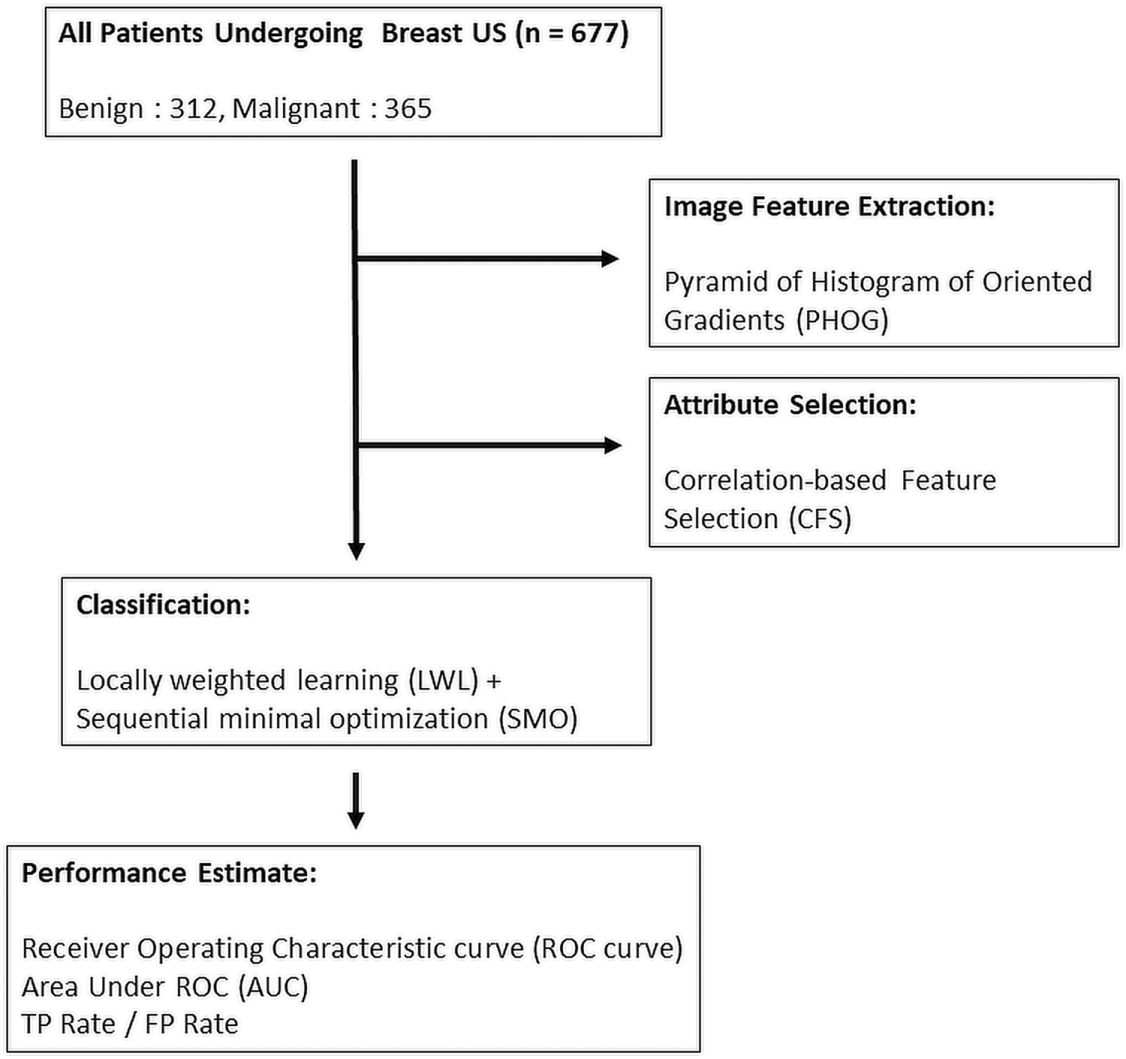
Flowchart of patient enrolment and the data analysis procedure.

The corresponding pathological and image reports for each US image of the participants were also collected. The image reports were used to obtain the BI-RADS category, and the pathology report was used as the gold standard for classifying all enrolled patients into benign or malignant categories. The identification of all solid masses on US images and the determination of the category they belonged to were based in the American College of Radiology (ACR) BI-RADS category criteria and were checked by experienced surgeons (> 10 years experienced in breast ultrasound). The sensitivity and specificity values associated with the physician diagnoses were also estimated and used for comparisons. A flowchart of the enrolment and data analysis procedure used in this study is shown in Fig. 1.

### Image feature extraction

**Figure 2:**
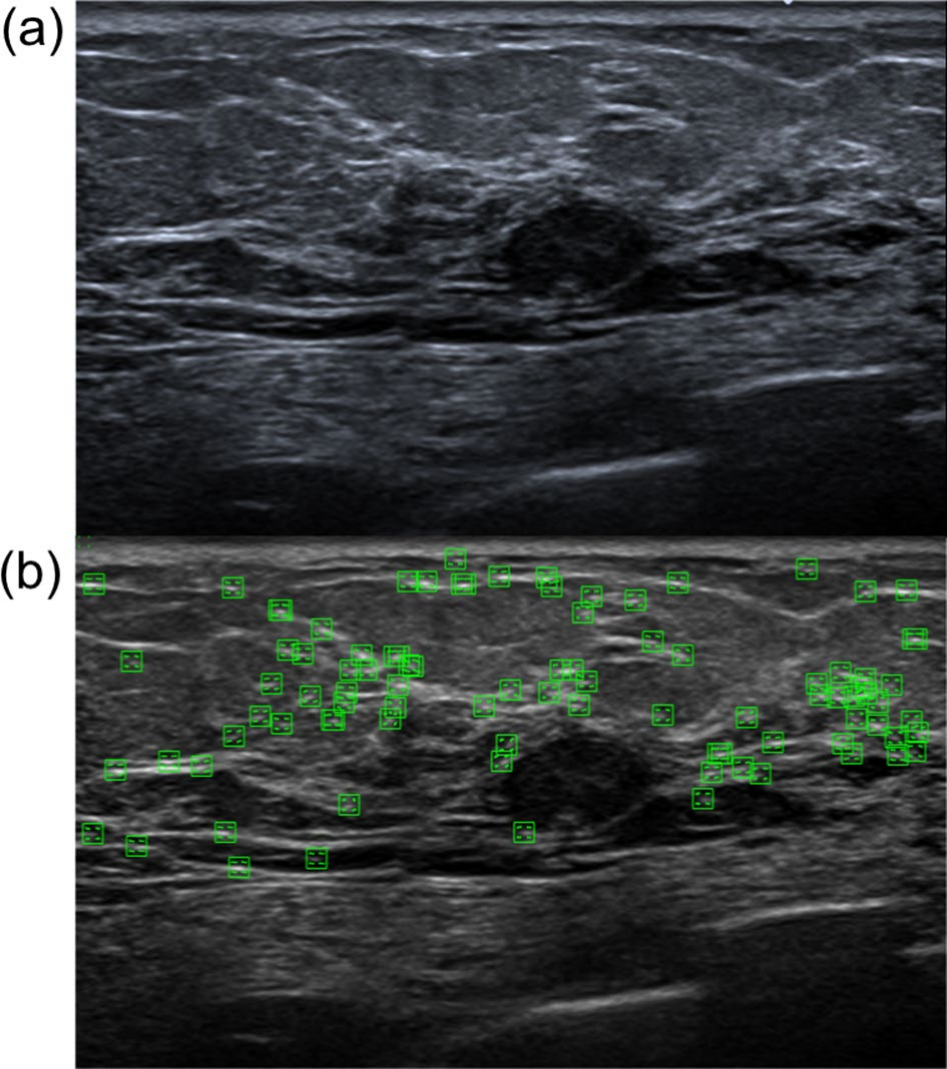
Visualised extracted feature vector from a benign US image by Histogram of Oriented Gradient (HOG). The probably benign (BI-RADS category 3) case: female, age 78 years, a left breast hypoechoic tumour was found and reported by ultrasound imaging, size: 11.7 × 6 mm, location: 2 o’clock from the nipple, distance: 2 cm from the nipple to the lesion. The fibroadenoma lesion was confirmed after a core needle biopsy, size: 1.2 × 0.1 × 0.1 cm. The extracted feature vectors through the HOG descriptor from the image are shown as corner points. (**a**) The US image of this case. (**b**) Green marks represent the positions of corner points obtained from the accelerated segment test (FAST).

**Figure 3:**
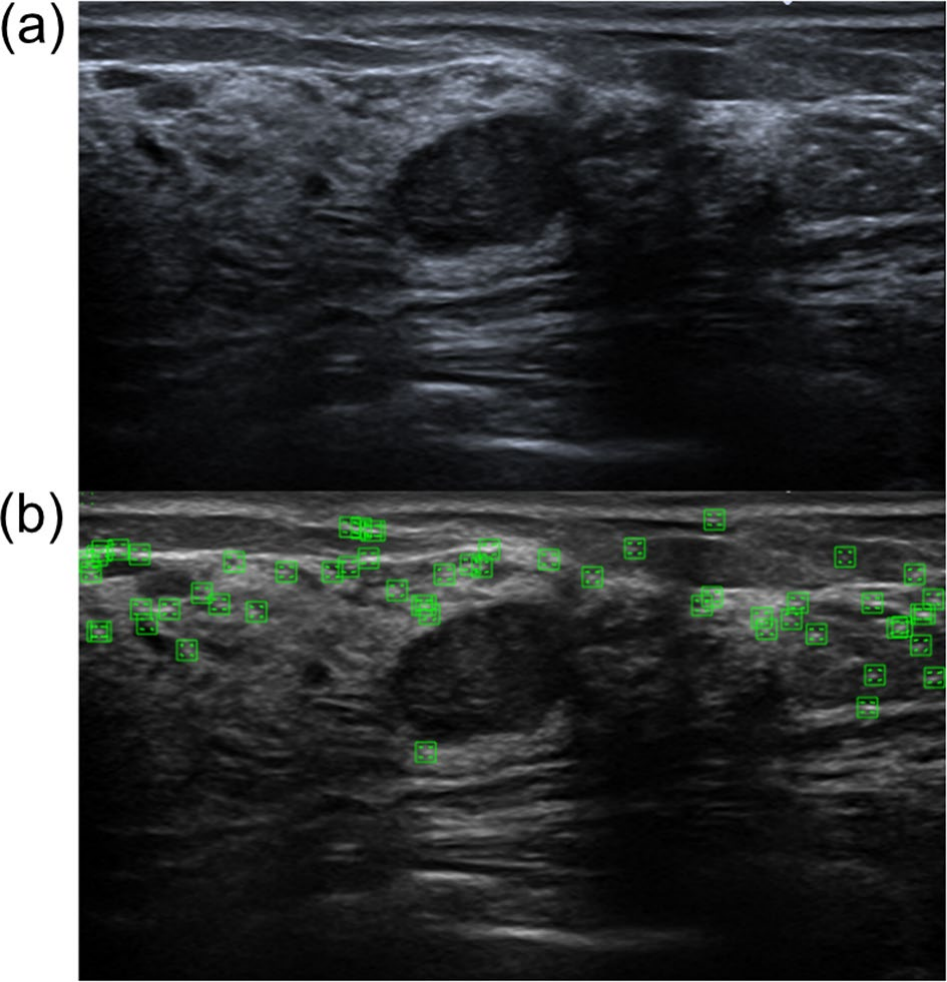
Visualised extracted feature vector from a malignancy US image by Histogram of Oriented Gradient (HOG). The moderate suspicion for malignancy (BI-RADS category 4B) case: female, age 51 years, an irregular and vascularised mass was found at the left breast and reported by ultrasound imaging, size: 1.2 × 0.9 cm, location: 1–2 o’clock from the nipple, distance: 6 cm from the nipple. A Core needle biopsy was performed and confirmed ductal carcinoma in situ. The infiltrating ductal carcinoma was confirmed after a partial mastectomy, size: 2.2 × 1.6 × 0.9 cm. The sentinel lymph node dissection was negative for malignancy at the left (0/2), and the immunohistochemical study of cytokeratin shows no metastatic carcinoma cells. The extracted feature vectors through the HOG descriptor from the image are shown as corner points. (**a**) The US image of this case. (**b**) Green marks represent the corner point positions obtained from the accelerated segment test (FAST).

The shape, edges, and corner points are important features in image classification. Among them, the corner points are generally considered the points with sharp change in grey level, or the high curvature points at the edge of the image. A possible strategy for image classification is to build a method that can represent the shape using the spatial distribution to facilitate recognition. Here, we used a traditional feature presentation describer method, named histogram of oriented gradients (HOG)^11^, to visualise this concept. The features from the accelerated segment test (FAST)^12^, which was based on HOG, were utilised to determine if important classification features could be extracted from preliminary US images. FAST is a corner detection method, and it was used to extract feature points and then track and map interesting objects on an image. The benefit of FAST is its performance, since it is faster than many other methods. The HOG was the feature descriptor; it was used to extract useful information and discard redundant information to simplify the subsequent image classification by calculating and counting gradient histograms of local areas of images. Figure 2 demonstrates how FAST based on HOG can be used in feature extraction to obtain appearance and shape descriptions from sample US images. The extracted features are shown as corner points. Figure 2a is a hypoechoic tumour US image that belongs to BI-RADS category 3 (the fibroadenoma was confirmed after core needle biopsy), and Fig. 3a is a US image of an irregular and vascularised mass that belongs to BI-RADS category 4B (the infiltrating ductal carcinoma was confirmed after a partial mastectomy, size: 2.2 × 1.6 × 0.9 cm). Two presentation US images were randomly selected from the dataset, and their BI-RADS categories were confirmed by an experienced physician. After FAST was applied to the probably benign (Fig. 2b) and moderate suspicion for malignancy (Fig. 3b) US images, it can be seen that the corner point in the malignant US image was located in the vicinity of the lesion. Thus, a comparison of the distribution of the corner points could help distinguish probably benign tumours from malignant tumours. This experiment confirmed that HOG may have the ability to allow preliminary discrimination between benign and malignant US images.

**Figure 4:**
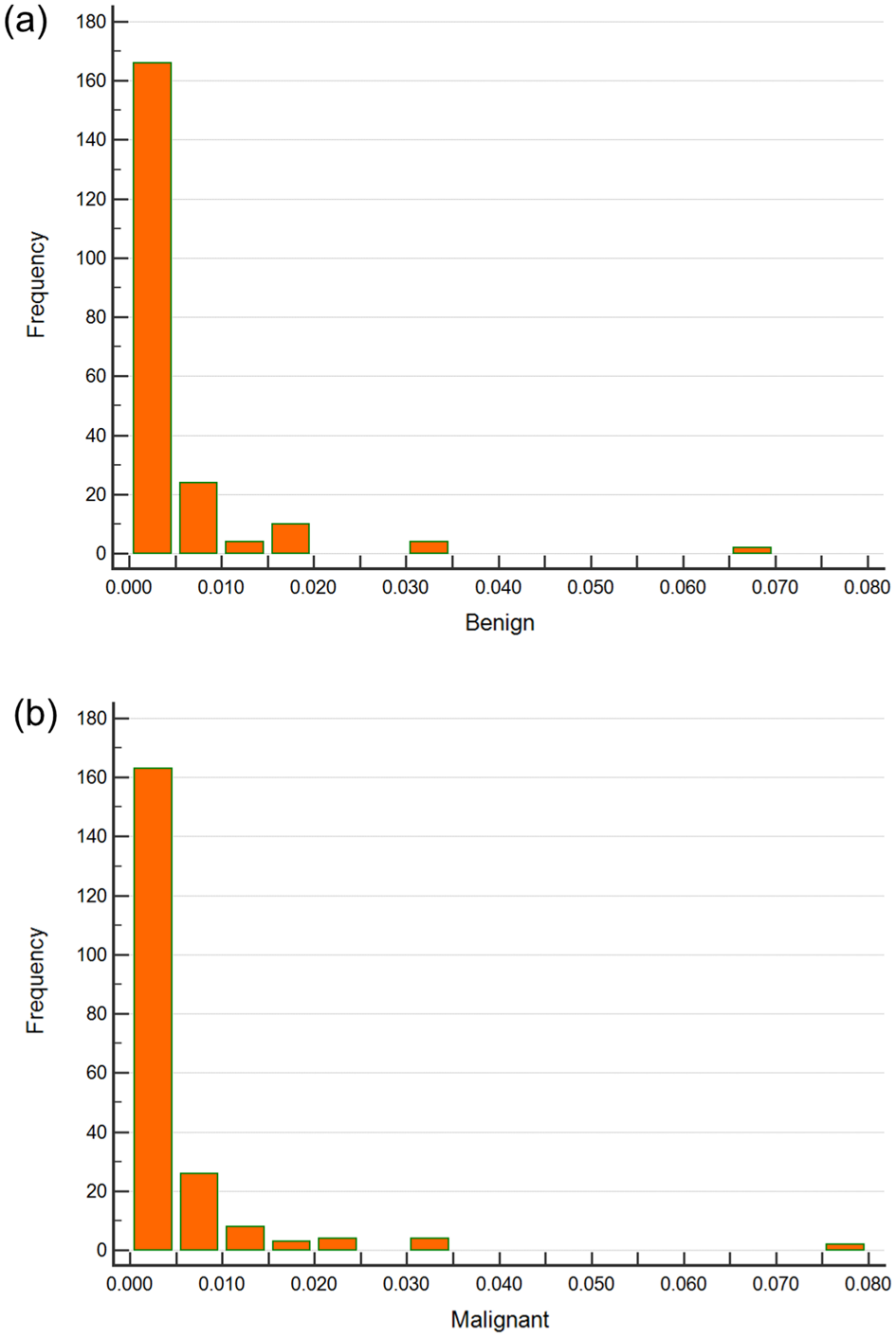
Histograms of PHOG Descriptors for Benign and Malignant Tumours in US Images. The histograms of PHOG descriptors that extracted local shape and the spatial layout from images and quantified them as vectors for (**a**) a probably benign US image (Fig. 2a, level = 2, bin = 30, total 630 features) and (**b**) a moderate suspicion for malignancy US image (Fig. 3a, level = 2, bin = 30, total 630 features).

To improve the performance of image classification, a newer method named the pyramid histogram of oriented gradients (PHOG)^13^ descriptor was used for the representation of shapes using the spatial distribution of US images in this study. This descriptor consists of HOGs associated with each image subregion at each resolution level and uses image pyramid representation^14^ to represent the local shape and the spatial layout of the image shape simultaneously. In comparison with HOG, it captures the spatial distribution of edges and is formulated as a vector representation. The distance between two PHOG image descriptors reflects the extent to which the images contain similar shapes and corresponds to their spatial layouts. Figure 4a, b show the histogram representing the distributions of the vector in the PHOG descriptor for two US images of benign and malignant tumours. To achieve better performance and avoid some of the disadvantages of HOG, canny edge detection^15^ was also applied to facilitate edge detection in the PHOG descriptor calculation for US images. The PHOG descriptor calculation and canny edge detection were implemented by calling the external library of the PHOG descriptor library from the lucene image retrieval (LIRE) project^16,17^ and integrated into MATLAB 2019a (The Math Works, Natick, MA, USA) for further use.

### Feature selection

The PHOG descriptor calculated the image level that contained similar shapes and exhibited similar spatial layouts. It may have hundreds of feature vectors according to the different parameters selected. Irrelevant and redundant feature vectors decrease the accuracy of the predictions and classifications, and selection of features that contain information important for classification and ruling out nonmeaningful descriptors were useful for further analyses and for speeding up the computation. In this study, a strategy named correlation-based feature selection (CFS)^18^ was used to evaluate the important attributes, and a subset was created while considering the predictive abilities along with the degree of redundancy. The evaluation function was utilised to evaluate subsets that contained features that were highly correlated with the class and uncorrelated with each other. Irrelevant features were ignored, and redundant features were screened out. For each subset, the best-first search method^19^ moves through the search space by making local changes to the current feature subset and selects useful subsets by backtracking along the search path. If the path being explored begins to look less promising, it can backtrack to the last subset that was more promising and continue the search. The search direction is bidirectional, and the search will be terminated when non-improving nodes are greater than four.

### Classification

In this study, the classification method was a combination of locally weighted learning (LWL)^20^ and sequential minimal optimisation (SMO)^21^. The LWL method is nonparametric, and the current predictions are obtained using local functions that employ subsets of data. LWL does not construct a global model of the entire functional space, but it instead creates a local model for each point of interest (POI) based on the data around a neighboring point. In this study, the K-nearest neighborhood (KNN) weighting function was utilised as the kernel function in LWL. Thus, identification of the K-nearest neighborhood in the training set based on the identification of the classified/regressive points of the test samples was achieved by (a) weighting their k-nearest neighbors in the training set and (b) weighting the contribution of each training point based on certain functions (kernels) of the distance between the test points and the training. For determining the optimum parameter for KNN, the SMO was used to solve the quadratic programming problem in optimisation by dividing the overall problem into several solvable sub-problems and ensuring convergence^22^.

### Diagnostic performance estimate of physicians

Owing to the nature of the retrospective study, images and corresponding reports of all enrolled patients were collected in advance. We did not repeat the human readout procedure for the estimation of diagnosis performance. After referring to the study design of recent related studies^23,24^, we modified the study design of the diagnostic performance estimate of physicians to fit this study. The image report of all participants was read and completed by four physicians (including one of the authors). Since these four physicians were all senior and having over 10 years of experience in breast US and diagnosis, and the acquisition procedure of breast US was also highly standardised in the institute, the correlation coefficient of the readout performance among these physicians was not estimated and considered as the total performance. All images were rated as benign or malignant according to the BI-RADS category (BI-RADS < 3: benign, BI-RADS > 4: malignant). this was similar to the traditional 3-point or 5-point Likert-type scale for malignancy of human readout in related studies (roughly corresponding to the BI-RADS classification with 5 meaning > 98% probability of breast cancer). It needs to be noted that the performance presented here does not represent the absolute ‘benign’ or ‘malignant’ classification of US images by human readout; it represents the judgement of biopsy that the physicians need to make during diagnosis.

### Computation platform

All computations were performed on an HP Z420 workstation equipped with an Intel Xeon E5 1620 CPU (Quad-Cores, the clock up to 3.6 GHz), 20 GB DDR3 ECC RAM, SanDisk 1 TB SATA 6 GB/s solid-state drives, Windows 10 professional edition, and a Nvidia Quadro K600 graphics processing unit (GPU) (equipped with 1 GB video memory). The whole computation did not rely on the accelerated graphics processing unit hardware because the intermediate data generated during computation were over the size of the internal video memory.

### Classification performance evaluation and statistical analyses

Ten-fold cross-validation was used to determine the error percentage, mean, standard deviation, and 95% level confidence interval for the baseline algorithms. The diagnostic accuracy was estimated using the area under the receiver operating characteristics (ROC) curve (AUC) and was compared with DeLong’s nonparametric test. Youden’s index^25^ was utilised to determine the optimal cut-off and the resulting specificity, sensitivity, positive predictive value (PPV), and negative predictive value (NPV). McNemar’s test^26^ was used to compare the sensitivities and specificities on a classification table. The statistical analyses were performed using MedCalc for Windows (Version 19.2.1, MedCalc Software, Ostend, Belgium). A p-value < 0.05 was considered indicative of significant differences.

## Results

### Characteristics of the image set

Table 1 presents the basic characteristics of all enrolled patients. In this study, after applying the exclusion criteria, there were 312 patients with solid masses, including fibroadenomas, and 365 patients with malignant tumours. All the enrolled patients also underwent pathological confirmation (either by fine-needle cytology, core-needle biopsy, or open biopsy), and 1354 US images were acquired. The mean age, mean lesion size, proportion of each BI-RADS category, and the tissue types in patients with benign and malignant masses are also listed. In benign cases, the most common tissue types of solid masses were fibroadenomas (78/312, 25.0%) and fibrocystic changes (105/312, 33.65%), and the incidences of lobular carcinoma in situ (LCIS) and fibroepithelial lesions were 4.49% (14/312) and 23.08% (72/312), respectively. For malignant tissue types, the incidence of ductal carcinoma in situ (DCIS) was 20.82% (76/365), and the most common tissue type was invasive ductal carcinoma (IDC) (76.25%, 289/365). After applying the PHOG descriptor calculation to extract the feature vectors, 630 attributes were extracted from each US image of the dataset, and 60 attributes were preserved after applying the feature selection. These filtered attributes were then sent to the classifier for classification.

**Table 1:** Characteristics of all enrolled patients.

Characteristics	Benign	Malignant
**Age of patients (years in mean ± SD)**	43.70 (42.23–45.18)	55.88 (54.39–57.38)
**Lesion size of US (cm in mean ± SD)**	1.56 (1.44–1.73)	2.28 (2.14–2.42)
**BI-RADS category: patients in percentage (%)**		
1	–	–
2	7 (2.24%)	–
3	63 (20.19%)	11 (3.01%)
4A	222 (71.15%)	130 (35.62%)
4B	14 (4.49%)	46 (12.60%)
4C	3 (0.96%)	43 (11.78%)
5	1 (0.32%)	106 (29.04%)
6	1 (0.32%)	29 (7.95%)
**Malignant tissue: patients in percentage (%)**		
DCIS	–	76 (20.82%)
IDC	–	289 (79.18%)
**Benign tumour: patients in percentage (%)**		
LCIS	14 (4.49%)	–
Fibroadenoma	78 (25.00%)	–
Fibrocystic change	105 (33.65%)	–
Intraductal papilloma	14 (4.49%)	–
Stromal fibrosis	5 (1.60%)	–
Fibroadenomatoid mastopathy	1 (0.32%)	–
Adenosis	6 (1.92%)	–
Fibroepithelial lesion	72 (23.08%)	–
Morphological features of carcinoma	17 (5.45%)	–

Basic characteristics of all enrolled patients and the proportions of benign (n = 312) and malignant (n = 365) cases include the age of patients (mean ± SD), size of lesions in the US and the BI-RADS category / benign and malignant tissue of patients. US: ultrasound; DCIS: ductal carcinoma in situ; LCIS: lobular carcinoma in situ; IDC: invasive ductal carcinoma; Morphological features of carcinoma: it showing morphological features of carcinoma in the US image, and confirmed to be benign tissue after a biopsy.

### Diagnosis performance

**Figure 5:**
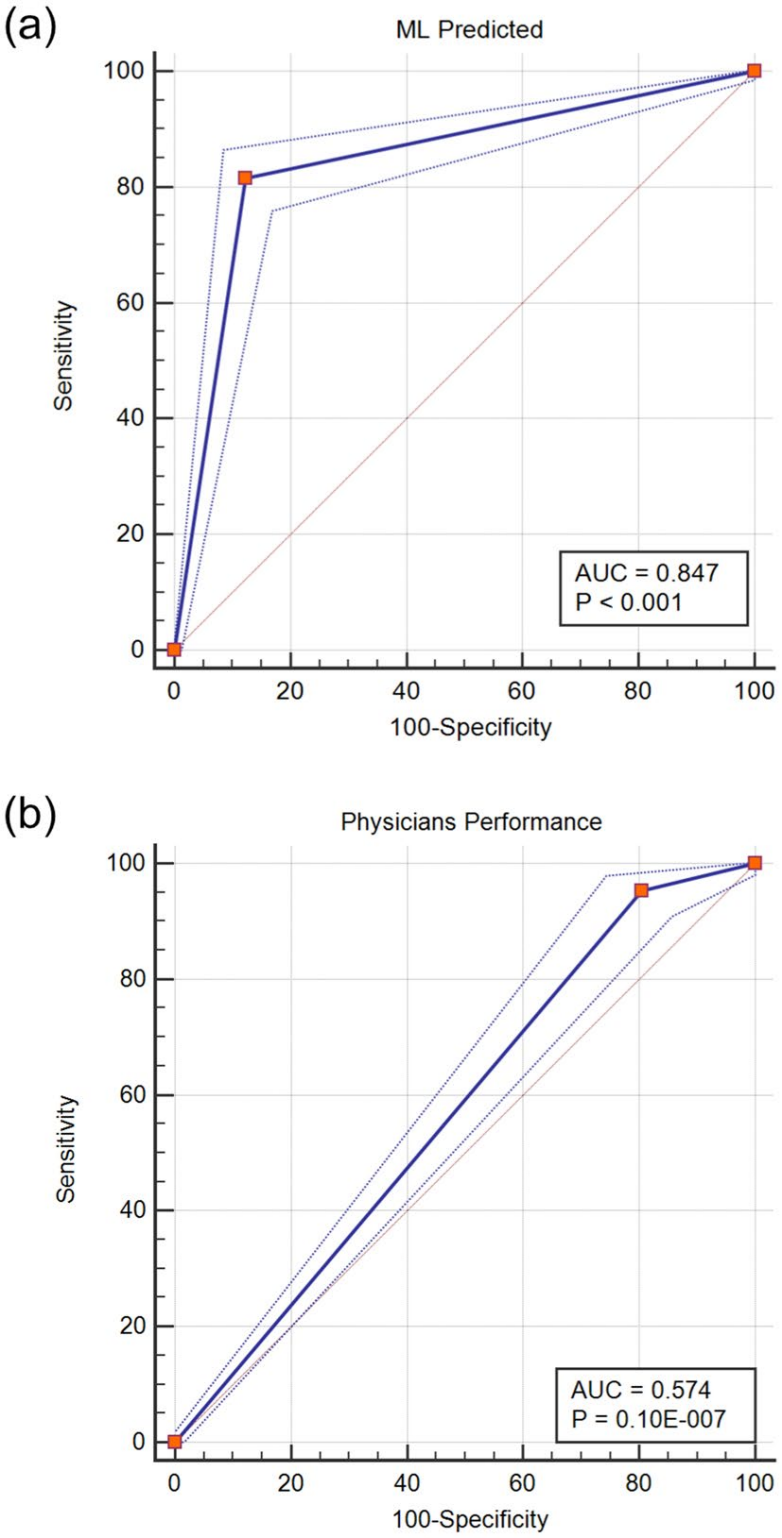
ROC Curves and the Classification AUCs Based on Machine Learning and Physician Diagnoses. (**a**) Classification using machine learning and (**b**) classification based on physician diagnoses.

In this study, the AUC of malignant and benign classifications by using unsupervised machine learning was 0.847 (SE = 0.819 to 0.872). The sensitivity was 81.64% and the specificity was 87.76% (p < 1 × 10^–5^). The PPV and NPV were 84.1 and 85.8%, respectively. The ROC curve and the AUC are shown in Fig. 5(a). Compared to the diagnostic performance of physicians, the AUC associated with the diagnoses by the physicians was 0.574 (SE 0.532 to 0.615); the sensitivity and specificity were 95.28 and 19.50%, respectively (p < 1.01 × 10^–8^), while the PPV and NPV were 48.2 and 84.0%, respectively. The ROC curve and the AUC are shown in Fig. 5(b).

## Discussion

This study proposes a new process flow that integrates existing machine learning algorithms in malignant/benign classification of US images. The whole image produces the features used in classification; therefore, the regions-of-interest (ROIs, i.e. tumour region) of each US image in the dataset do not require preselection. This makes data processing and implementation more concise while ensuring similar or improved performance. This outcome is preliminary— yet indicative—of machine-learning performance. It also achieves unsupervised learning and presents potential applicability regarding clinical diagnoses.

Recently published articles and utilised approaches related to this topic were also discussed and compared, to confirm that this study contributes to this field. We compared 11 articles related to this topic published during 2015–2020, based on a similar premise. Owing to the different methodology utilised and some common issues in these studies, the classification performance cannot be compared with our study directly: (1) In the present study, the full scan plane of breast US was utilized as input, and no tumour labelling procedure before classification (include labelling tumour region or tumour contour). In previous studies, the selection of the ROIs (i.e., the tumour regions) was defined manually by physicians. (2) Less number of cases in benign and malignant lesions in some studies (usually less than 200 cases). (3) the image dataset was based on the public dataset, or lack tissue-proof for benign masses or malignant lesions in some studies. Table 2 lists these references and related issues. The greatest limitation of these studies was that they required the ROI pre-selection before the classification, and the correct image feature generation was heavily dependent on this step. Thus, differences in ROI selection affected the outcome of CAD, resulting in variability among observers when physicians joined the process. Large datasets require a considerable amount of work, leading to biases among observers, which is to be avoided. In this study, this process flow omits complex tumour segmentation procedures or artificial ROI selection and the features utilised to recognise benign or malignant US images were generated and selected automatically. This decreased the human effort significantly and made automated CAD possible.

**Table 2:** Summary of articles in classification of breast tumour.

References	Topic	Modality	Strategies	Sample Size	Performances	Issues
Choi et al.^34^	Effect of a deep learning framework-based computer-aided diagnosis system on the diagnostic performance of radiologists in differentiating between malignant and benign masses on breast ultrasonography	US	1. Deep Learning (GoogLeNet)	173 benign and 80 malignant breast messes	Sensitivity: 85.0% Specificity: 94.0%	1, 2
Zhou et al.^31^	Classification of benign and malignant breast tumours in ultrasound images with posterior acoustic shadowing (PAS) using half-contour features	US	1. Disk expansion (DE) segmentation 2. Half-contour detection of PAS: tumour circularity (TC) and standard deviation of degree (SDD)	40 cases with PAS / 10 cases without PAS	Sensitivity: 72% Specificity: 76% AUC: 0.78	
Becker et al.^23^	Classification of breast cancer in ultrasound imaging using a generic deep learning analysis software	US	1. Deep learning-based image software (ViDi Suite v 2.0)	82 patients with malignant / 550 with benign lesions	Sensitivity: 80.4% Specificity: 84.2% AUC: 0.84	1
Ciritsis et al.^32^	Automatic classification of ultrasound breast lesions using a deep convolutional neural network	US	1. Deep Learning (dCNN)	40 patients	Sensitivity: 92.1% Specificity:76.0% AUC: 83.8	1
Byra et al. ^38^	Classification of breast lesions using segmented quantitative ultrasound maps of homodyned K distribution parameters	US	1. Homodyned K distribution maps 2. Markov random field model	32 malignant breast tumours / 71 benign masses	Sensitivity: 76.8% Specificity: 75.8% AUC: 0.84	2, 3, 6
Silva et al.^29^	Breast tumour classification in ultrasound images using neural networks with improved generalisation methods	US	1. Neural network 2. 22 mixed features	50 malignant and 50 benign tumours	Sensitivity: 97% Specificity: 96% AUC: 0.98	2, 3
Cai et al. ^39^	Robust phase-based texture descriptor for classification of breast ultrasound images	US	1. Phase congruency detection 2. Texture-based feature extraction 3. SVM	69 benign and 69 malignant cases	Sensitivity: 83.4% Specificity: 85.4% AUC: 0.86	2, 3
Ara et al.^27^	Bimodal multiparameter-based approach for benign–malignant Classification of breast tumours	US / UE	1. Genetic Algorithm	170 patients (56 malignant lesions and 145 benign cases)	Sensitivity: 89.3%* Specificity: 80.1%* AUC: 0.91*	2, 3
Moon et al. ^40^	The adaptive computer-aided diagnosis system based on tumour sizes for the classification of breast tumours detected at screening ultrasound	US	1. Quantitative morphological and textural features 2. Linear logistic regression	156 tumours (78 benign and 78 malignant)	Sensitivity: 85.4% Specificity:77.8% AUC: 0.86	2, 3
Singh et al. ^30^	Fuzzy cluster based neural network classifier for classifying breast tumours in ultrasound images	US	1. Fuzzy c-means clustering / back-propagation artificial neural network 2. SVM	178 patients and mixed with open data (88 benign / 90 malignant)	Sensitivity: 94.7% Specificity: 93.6% AUC: 0.94	2, 3, 5, 6
Jain et al. ^28^	Texture ratio vector technique for the classification of breast lesions using SVM	US	1. Texture feature: inside the lesion (IAI) and upper side of lesion (UAI) 2. Statistical texture: 7 features 3. SVM	117 images (45 benign and 72 malignant) from open data	Overall classification accuracy: 86.6%	2, 3, 5, 6

US: ultrasound, UE: ultrasound elastography, CDUS: colour-Doppler flow imaging ultrasound, SMI: superb microvascular imaging ultrasound.

*: Report the performance using B-mode US image only.

Issues: ^1^ Not based on machine learning strategies^2^, Using semi-segmentation or contour pre-processing to labelling the tumour region^3^ , Feature extracted from only partial US image^5^, Unspecified all cases were tissue-proved^6^, Unspecified ultrasound system model/manufacturer.

Some studies in Table 2 also present higher sensitivity/specificity or AUC than our study. The area under the curve (AUC) of the ROC regarding the distinction between benign and malignant tumours was approximately 0.86–0.9^27–30^. However, due to quality defects of image dataset (i.e. fewer participants, unspecific ultrasound system/model or using open data and lack the tissue-proof of masses or lesions, etc.) in these studies, it makes these results may lack of representative. The common point of these studies was using the various feature-detection to simultaneously pre-select ROIs and shows that one or two features in these detections were useful in malignant classification after experimentation; however, when processing diverse variations of US images in a considerably large dataset, it is uncertain which one—or which combination—of the features should be utilised to promote classification in different situations. Singh et al.^30^ and Silva et al.^29^ combined the neural network/back-propagation artificial neural network or SVM for the fuzzy classification from various extracted image features and showed good classification performance in preliminary studies. This method will potentially improve this study’s performance in the future.

Preventing the disadvantage of manual pre-selection of ROIs (i.e. considerable human effort) still relies on the analysis of tumour region/contour to the extraction features; automatic segmentation is a possible method to overcome this problem; however, an error-free tumour region selection is difficult and limited by image quality. Even an effective automated segmentation algorithm would require multiple rounds of fine-tuning, increasing implementation complexity. Zhou et al.^31^ used disk expansion segmentation by detecting the variant of posterior acoustic shadowing to implement the automatic segmentation of the ROI from the full scan plane of a US image, and then complete the classification of malignant lesions. The classification’s sensitivity and specificity were 72% and 76%, respectively. The developments and advances in deep convolutional neural networks also raised the expectations for fully automatic feature extraction for breast lesion classification in ultrasound images. The average performance of classification in recent publications regarding sensitivity and specificity were 0.86 and 0.85, respectively^23,32–34^.

Regarding the comparison of the diagnostic performance between machine learning and physicians, the estimation of diagnostic performance by the human readout procedure was omitted in this study, but the performance was calculated from the reported image and the pathology report, according to the BI-RADS category for direct judgment. Although the diagnostic performance of using unsupervised machine learning approaches in this study is better than that of physicians, we still lack the human readout performance as a comparative criterion. Here, recently published articles can provide this information and be used for reference. A published article reported that the AUC of malignancy detection by B-mode US was only 0.698 in differentiating BI‑RADS 4 breast lesions^35^. For the human readout performance of malignant lesions classification in US image, the AUC from several previous studies and using similar estimated methods was between 0.6 and 0.91, and PPV (from 0.46 to 0.81) and sensitivity (47–85%) varied widely. The wide ranges in the concordance correlation coefficient between readers (from 0.21 to 0.71)^23,36,37^ also presents the inconsistent and bias between intra-observers. In this study, the performance of this unsupervised classification was similar and even better than that of the participating physicians. Even though the entire process did not involve physicians, the classification performance was not inferior to those reported in previous studies.

The main limitation of this study was the variability in the expertise levels of the participating physicians. Therefore, biases associated with the observers cannot be ruled out. The extracted malignant features were obtained by the PHOG descriptor, which is not synonymous and does not map to the BI-RADS lexicons. This also represents a clinical limitation of the application. Moreover, classification training could be improved if there were more enrolled patients and larger US image datasets since this would increase classification accuracy and lead to fewer false-positive and more true-negative outcomes.

Concluding, the method and procedure presented in this study used machine learning to predict whether tumours were benign or malignant based on US images. The findings showed that the performance outcomes were similar to those obtained with assessments by physicians. The use of machine learning in the analysis of US images may help improve the diagnostic capacity of radiologists by providing “second opinions” on the classification of unknown benign and malignant breast tumours in US images. This ultimately minimises the effort expended by physicians to make diagnoses based on image analysis.

## Data Availability

The datasets generated during and analysed during the current study are not publicly available due to IRB and institutional restrictions, but are available from the corresponding author on reasonable request.
